# The functional effects of Piezo channels in mesenchymal stem cells

**DOI:** 10.1186/s13287-023-03452-y

**Published:** 2023-08-26

**Authors:** Zhilong Huang, Yingying Huang, Xiner Ning, Haodi Li, Qiqi Li, Junjie Wu

**Affiliations:** State Key Laboratory of Military Stomatology and National Clinical Research Center for Oral Diseases and Shaanxi Clinical Research Center for Oral Diseases, Department of Orthodontics, School of Stomatology, The Fourth Military Medical University, Xi’an, 710032 China

**Keywords:** Piezo1, Piezo2, Mechanosensitive ion channels, Mechanotransduction, Mesenchymal stem cells

## Abstract

Mesenchymal stem cells (MSCs) are widely used in cell therapy, tissue engineering, and regenerative medicine because of their self-renewal, pluripotency, and immunomodulatory properties. The microenvironment in which MSCs are located significantly affects their physiological functions. The microenvironment directly or indirectly affects cell behavior through biophysical, biochemical, or other means. Among them, the mechanical signals provided to MSCs by the microenvironment have a particularly pronounced effect on their physiological functions and can affect osteogenic differentiation, chondrogenic differentiation, and senescence in MSCs. Mechanosensitive ion channels such as Piezo1 and Piezo2 are important in transducing mechanical signals, and these channels are widely distributed in sites such as skin, bladder, kidney, lung, sensory neurons, and dorsal root ganglia. Although there have been numerous studies on Piezo channels in MSCs in recent years, the function of Piezo channels in MSCs is still not well understood, and there has been no summary of their relationship to illustrate which physiological functions of MSCs are affected by Piezo channels and the possible underlying mechanisms. Therefore, based on the members, structures, and functions of Piezo ion channels and the fundamental information of MSCs, this paper focused on summarizing the advances in Piezo channels in MSCs from various tissue sources to provide new ideas for future research and practical applications of Piezo channels and MSCs.

## Background

A significant class of mechanical transducers known as mechanically activated ion channels can efficiently convert mechanical stimuli into electrochemical signals that are essential for physiological and pathological processes [1–3]. A new era in mechanotransduction research began in 2010 with the identification of the proteins Piezo1 and Piezo2, which form mechanosensitive cation channels [4]. Mammalian Piezo1 and Piezo2 are an evolutionarily conserved class of large membrane proteins consisting of 2500 to 2800 amino acids [5–7]. Piezo1 and Piezo2 are widely distributed in various tissues in the human body (Table 1) [7–12]. In response to mechanical stimulation, Piezo channels are opened, allowing cations to cross the membrane and activate cells, and thus, these channels are involved in many physiological and pathological processes [4, 7, 13–16].

**Table 1:** The characteristics of Piezo1 and Piezo2

Items	Piezo1	Piezo2	References
Structure	A homotrimeric structure resembling a three-bladed propeller		[8, 11, 16]
Conducting cations	Selective	Nonselective	[4, 8]
Tissue distribution	Skin, bladder, lung, kidney, endothelial cells, periodontal ligament cells, erythrocytes, trigeminal sensory neurons, dorsal root ganglion	Trigeminal sensory neurons, dorsal root ganglion, Merkel cells, and somatic neuron cells	[7–12]
Function	Involved in mechanotransduction in a variety of cells	Senses slight touch and proprioception	[8–10, 13, 15, 17–20]
Activator	Yoda1, Jedi1/2	Not found yet	[8, 21, 22]
Inhibitor	Ruthenium red, gadolinium, streptomycin, and GsMTx4	Ruthenium red, gadolinium, streptomycin, GsMTx4, and FM1-43	[4, 7, 8, 23–25]

Mesenchymal stem cells (MSCs) have received extra attention for their abilities to promote tissue cell renewal and respond to tissue injury [26]. MSCs can be isolated from a wide range of tissues and can differentiate into many different tissue cells including chondrocytes, osteoblasts, muscle cells, cardiomyocytes, and blood cells. MSCs play a crucial role in wound healing, growth, and daily replacement of cells lost due to exfoliation or pathological conditions [27].

Because Piezo ion channels are stably expressed in MSCs, an increasing number of studies have shown that Piezo ion channels play an essential role in several physiological functions of MSCs. In recent years, it has been reported that the physiological processes of MSCs, such as proliferation, migration, osteogenic differentiation, adipogenic differentiation, angiogenesis, and osteoclast formation, may be regulated by Piezo channels [28–33]. Moreover, there have been many new findings on the molecular mechanisms by which Piezo channels affect the physiological functions of MSCs through cell signaling pathways. In addition, according to the biological mechanism by which Piezo channels affect MSCs, some scholars have designed instruments that can be used in the clinical treatment of diseases, promoting the translation from theoretical achievements to practical applications [30, 34–37]. However, no review has systematically classified and summarized these achievements in recent years. Therefore, based on the members, structures, and functions of Piezo ion channels and essential information on MSCs, this paper focused on the research progress on Piezo channels in MSCs from different tissue sources and mainly summarizes the possible role of Piezo channels in the physiological function of MSCs and its molecular mechanism. This review provides new ideas for future studies and practical applications of Piezo channels and MSCs.

## Piezo channels

### Piezo channels as biomechanical receptors for cells

How cells process mechanical signals and how these processes give rise to downstream signaling events are necessary to control cell fate. Piezo channels are among the most critical biomechanical sensors in cells. The pore-forming role of Piezo proteins in excitatory mechanosensitive ion channels was discovered for the first time in 2010 [4]. There are two types of Piezo channels in vertebrates: Piezo1 and Piezo2, which correspond to the encoding genes Fam38A and Fam38B, respectively [4], and are activated by stress. These proteins have been demonstrated to trigger cation currents and are mechanically activated in a wide range of eukaryotic cell types, thereby establishing a link between mechanical forces and cellular signals [5]. Piezo channels enable the cell to detect force by allowing positively charged ions, such as calcium ions, to flow into the cell in response to mechanical stimuli [11].

**Fig. 1:**
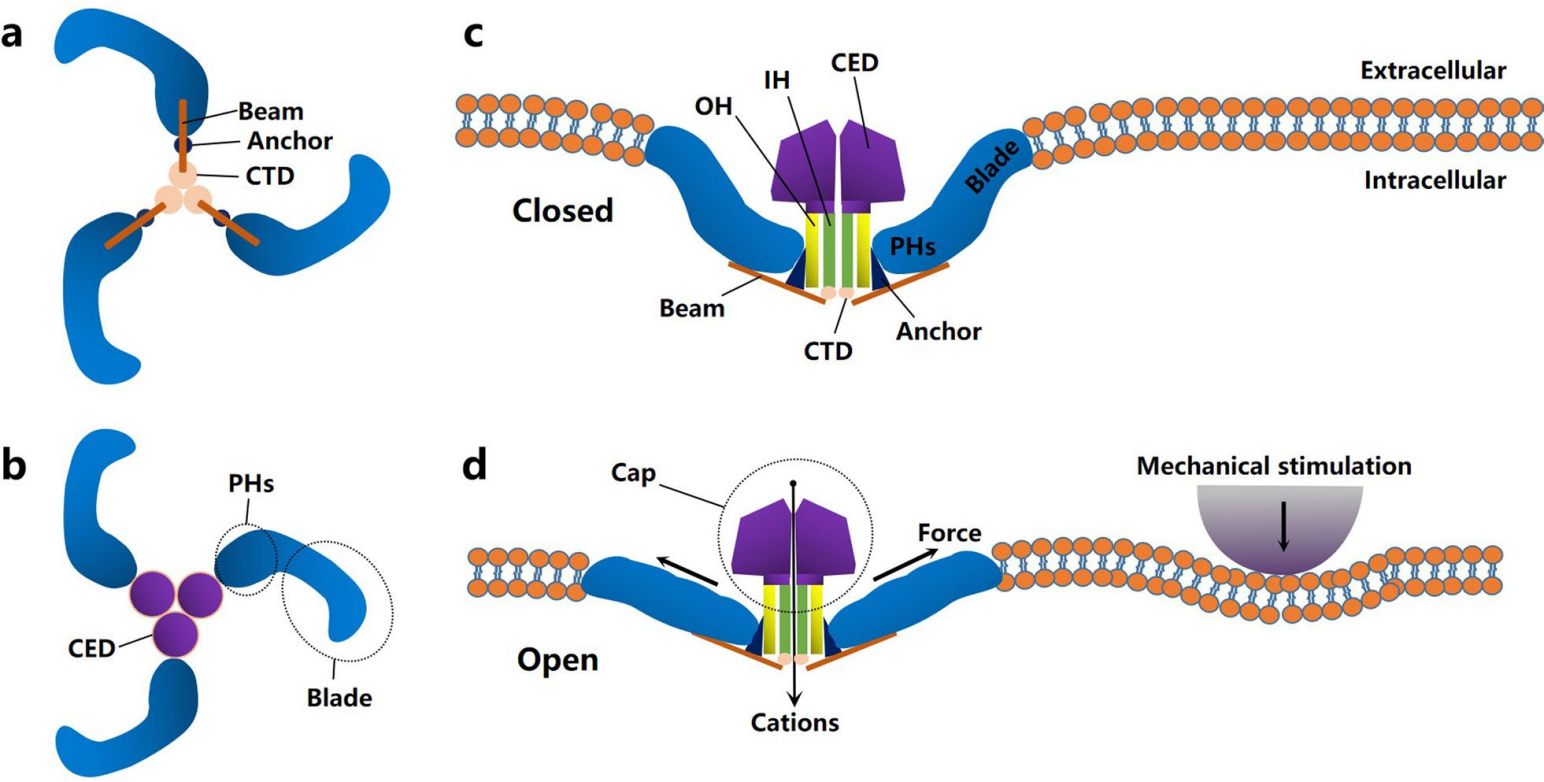
Schematic representation of the Piezo1 channel. **a** Bottom view; **b** top view; **c**, **d** lateral view. In response to mechanical stimulation, these arms bend, resulting in localized deformation of the cell membrane and opening of the channel. The cations then flow into the cytoplasm through Piezo channels, and the cells become activated, resulting in the corresponding biological effects. CED: C-terminal extracellular domain; CTD: intracellular C-terminal domain; IH: inner helix; OH: outer helix; PHs: peripheral helices. The figure was created for this article, and the image depicted in this figure is ours; it is not based on any previously published image

The 3D structure of Piezo channels reveals how proteins use various mechanical stimuli to activate the channel [38–40]. As shown in Fig. 1, the shape of the Piezo1 protein resembles a three-propeller structure consisting of three curved arms surrounding a central pore with an extracellular cap [8]. Previous studies have shown that in response to mechanical stimulation, the arm bends to cause local deformation of the cell membrane and the formation of specific protein–lipid interactions that affect the local morphology and composition of the bilayer. Finally, the cations flow into the cytoplasm through Piezo channels, and the cells become activated, thus producing the corresponding biological effects [8, 16, 40–42]. However, we still lack an understanding of the detailed gating mechanisms of these channels, and further studies are needed in the future.

### Piezo channels coupled to mechanical transduction

To facilitate the development of tissue engineering, it is essential to explore mechanical sensors in cells. Based on previous studies, Piezo channel-coupled mechanical transduction mechanisms can be grouped into four types. First, tension and shear stress on the cell membrane directly activate Piezo channels and cells. Second, direct mechanical cell-to-cell interactions activate Piezo channels. Third, Piezo channels are activated through the close involvement of extracellular matrix (ECM) components. Fourth, the cell is activated by the interaction between the intracellular cytoskeleton and the Piezo protein [43]. These mechanisms can cooperate and participate closely to complete mechanical transduction.

Piezo channels act as mechanosensors and are responsible for translating perceived extracellular mechanical stimuli into biochemical signals generated by the ions entering the pore. The electrochemical signal then gives rise to a series of intracellular downstream signaling pathways [16, 44] that control multiple aspects of cell proliferation and differentiation [43]. Furthermore, in addition to the mechanical activation of Piezo channels, it has been discovered that Yoda1 and Jedi1/2 activate Piezo1. Ruthenium red, gadolinium, streptomycin, and GsMTx4 inhibit Piezo1 and Piezo2 channels, and activators of Piezo2 have not been reported [4, 7, 21–23, 45] (Table 1).

### *Piezo channels involved in cytosolic Ca*^*2*+^*signaling*

Ca^2+^ is an intracellular signal that plays a critical role in cell regeneration and is a potent effector of cellular responses [46]. It can regulate protein activity and cell differentiation by inducing specific cellular signaling and transient transduction of information [47]. These properties emphasize the contribution of Ca^2+^ signaling events to cellular physiology, including tissue regeneration. Among the various Ca^2+^ signaling regulators, mechanical stress is one of the main effectors. Although the mechanisms of force perception and coupling remain unclear, mechanical forces are determinants of intracellular Ca^2+^ signaling. Piezo1 selectively conducts cations such as K^+^, Na^+^, Ca^2+^, and Mg^2+^ and has a slight preference for Ca^2+^. In contrast, Piezo2 has nonselective cation conductivity [4]. When Piezo channels are opened or activated, Ca^2+^ influx triggers cellular signaling cascades [16, 48–51]. For example, the Piezo-knockout phenotype in stem cells from the adult Drosophila midgut can be rescued by increasing the cytoplasmic Ca^2+^ concentration [52].

We can therefore conclude that Piezo channels play a key role in cellular signaling by converting mechanical signals into biochemical signals. Piezo channels act mainly through the Ca^2+^ signaling pathway [52]. Piezo channel opening leads to Ca^2+^ entry into the cell, and increased intracellular calcium ions act as second messengers, which can trigger the intracellular Ca^2+^ signaling pathway and regulate cellular functions [53].

### Piezo channels interacting with the cytoskeleton

Nourse and Pathak [54] found that Piezo channels could be gated by cytoskeletal pegging or force transduction through the membrane. Several studies have shown that Piezo1 is associated with lipid tension [42, 55]. In addition, Piezo2 regulates the formation of actin-based stress fibers and the orientation of adherent spots in a complex manner [56]. Furthermore, it has been observed that Piezo1 and the cytoskeleton have dynamic connections, and dynamic relationship between Piezo1 and the cytoskeleton has been reported [57]. For example, integrins are one of the main components of mechanotransduction linked to Piezo channels. The integrins of MSCs are considered direct mechanoreceptors that physically connect the ECM to the cytoskeleton and thus function as signaling receptors [58–60].

## The functional effects of Piezo channels in MSCs

MSCs are derived from a wide range of sources and have the capacity for multidirectional differentiation. MSCs play an important role in immune regulation, hematopoiesis, and tissue repair [61]. Researchers initially identified and isolated MSCs from cultured mouse bone marrow cells in vitro [62]. At present, MSCs have been isolated from several kinds of tissues, including bone marrow, adipose, synovial, perivascular, umbilical cord blood, umbilical cord tissue, placenta, menstrual blood, and dental pulp [61], and adipose tissue and bone marrow are the most commonly used sources of MSCs [63]. In addition, MSCs can be induced to differentiate into ectodermal cells (e.g., epithelial cells, neurons, and glial cells), mesodermal cells (e.g., adipocytes, chondrocytes, and osteocytes), endodermal cells (e.g., intestinal epithelial cells, myocytes, and lung cells), and cancer-associated fibroblasts [64–66]. Due to the multiple advantages of MSCs, such as pluripotency and self-renewal, MSCs are often used as primary functional cells in tissue engineering, regenerative medicine, and cell therapy [67, 68].

The cellular microenvironment consists of components that directly affect the surrounding environment of a cell or a group of cells, including cytokines, ECM, hormones, and surrounding cells. Additionally, there are mechanical forces from the surrounding environment. These forces exert significant effects on cell behavior through biochemical, biophysical, or other means [64]. Similarly, interactions with the microenvironment regulate the differentiation and proliferation of MSCs [69]. Specifically, MSCs can regulate the microenvironment, including the regulation of immunity [70–73], hematopoiesis [74], and tissue repair and regeneration [75]. The microenvironment can also influence the function of MSCs, and the mechanical signals provided to MSCs by the microenvironment have a particularly pronounced effect on their physiological functions, including osteogenic differentiation [76], chondrogenic differentiation [77], and aging [78]. In addition, MSCs interact with the mechanical microenvironment and influence disease progression in a variety of diseases, including tumors [79], rheumatoid arthritis [80], intervertebral disk degeneration [81], pulmonary fibrosis [82], and other diseases. In fact, Piezo channels are an important way for MSCs to sense mechanical stimuli [83].

### Piezo channels in bone marrow-derived MSCs

Sugimoto et al. [28] showed that Piezo1 acted as a hydrostatic pressure (HP) receptor in bone marrow-derived MSCs (BMSCs) and could promote the differentiation of BMSCs into osteoblasts while inhibiting BMSCs differentiation into adipocytes. Among mechanosensory receptors, Piezo1 is preferentially expressed in MSCs. BMP2 is an important growth factor for differentiating MSCs into osteoblasts [84, 85]. HP may activate the extracellular signal-related kinase 1/2 (ERK1/2) and p38 mitogen-activated protein kinase (MAPK) signaling pathways through Piezo1, which induces BMP2 expression [28]. This, in turn, upregulates Runx2 and Osterix expression, which initiates osteogenic genes such as ALP and COL1A1, thereby promoting osteoblast differentiation [28]. These results suggest that Piezo1 may play a role in determining the fate of BMSCs by regulating the expression of BMP2. Because aging and osteoporosis are accompanied by a relative decrease in osteoblastogenesis and a relative increase in adipogenesis, elucidating the molecular mechanisms that control the balance between osteoblastogenesis and adipogenesis is vital for improving therapeutic strategies for bone diseases. These findings provide important insights into the role of Piezo1 as a target for bone diseases. Researchers showed that 0.01 MPa HP was suitable for inducing MSCs to differentiate into osteoblasts [28]. However, different optimal pressure conditions may exist for MSCs to differentiate into different cell types, which needs further exploration. In addition, further studies are required to determine how Piezo1 is affected by intermittent or continuous HP and mediates the differentiation of MSCs.

In addition to HP, Wu et al. [29] found that the level of Piezo1 expression in BMSCs increased with increase in matrix hydrogel viscoelasticity, thus regulating the differentiation of BMSCs from osteogenesis to lipogenesis. Hydrogels can be administered with minimal invasiveness and are highly similar to living tissue. A study provided a practical idea to modulate the local microenvironment for tissue regeneration by modulating the viscoelasticity of hydrogels rather than just optimizing carriers for bioreagent delivery.

Similar to the results of these studies, Unnithan et al. [36] found that graphene oxide-based nanocomposites (GO-MNPs) functionalized with Piezo1 antibodies could activate Piezo1 and enhance ALP activity and calcium deposition in MSCs, thus enhancing osteogenic differentiation. This finding highlighted that Piezo1 activation was likely associated with the osteogenic differentiation of MSCs.

A study by Wang et al. [30] used triboelectric stimulation generated by a wearable pulsed triboelectric nanogenerator (WP-TENG) to increase the intracellular Ca^2+^ concentration by activating the mechanosensitive ion channel Piezo1. This, in turn, upregulated the expression of the osteogenesis-related genes Col1a, Runx-2, and OCN and increased the expression of hypoxia-inducible factor-1α (HIF-1α) and the angiogenic factors endothelin 1 (EDN1) and vascular endothelial growth factor A (VEGFA), which ultimately rejuvenated aging BMSCs and enhanced osteogenic differentiation and proangiogenic functions [30]. Another study showed that the recognition of periodic forces by Piezo1 drove the stabilization and transcriptional upregulation of HIF-1α [86]. HIF-1α promotes osteogenesis and bone defect repair in BMSCs by enhancing the expression and secretion of the downstream osteogenesis-related genes EDN1 and VEGFA [87]. However, if HIF-1α expression is inhibited, this effect is not observed even when Piezo1 is activated. These results suggest that HIF-1α is a key signaling factor in the Piezo1-related signaling pathway that promotes the rejuvenation of aged BMSCs. In addition, another study [88] used pulsed triboelectric stimulation generated by P-TENG to rejuvenate senescent BMSCs by enhancing murine double minute 2 (MDM2)-dependent p53 degradation. This effect was confirmed in loss-of-function studies of MDM2 and p53. In the two studies, the stimulation of senescent BMSCs was pulsed triboelectric stimulation generated by P-TENG, and we hypothesized that in addition to HIF-1α acting as a key factor in the Piezo1-related signaling pathway to promote the rejuvenation of aged BMSCs, the MDM2-p53 pathway also plays a significant role. Overall, activation of Piezo1-related signaling pathways can rejuvenate aging BMSCs, enhance their osteogenic differentiation, and promote angiogenesis. However, the current research results in this area are limited, and more detailed and specific signaling pathway mechanisms remain to be elucidated. For practical purposes, the potential future clinical use of WP-TENG to activate Piezo1 may involve treating osteoporotic fractures and repairing and regenerating bone defects after internal or external fixation.

Tsimbouri et al. [37] found that 3D osteogenesis by nanovibrational stimulation in BMSCs is a mechanotransduction process involving intracellular tension, and mechanoreceptors such as Piezo, TRP, and KCNK are involved. In another study on the nanomechanical stimulation of BMSCs, Orapiriyakul et al. [35] found that a certain intensity of nanovibrational stimulation could convert BMSCs into osteoblasts in two and three dimensions, which could be related to Piezo1/2 and its downstream target ERK1. Ambattu et al. [89] showed that in human MSCs from various donor sources, including human bone marrow-derived MSCs (hBMSCs), adipose-derived stem cells (hADSCs), and umbilical cord blood-derived stem cells (hUCSCs), short-duration high-frequency megahertz-order nanomechanostimulation induced the differentiation of MSCs into the osteoblast lineage by significantly upregulating early osteogenic markers such as RUNX2 and COL1A1 and increasing late markers such as osteocalcin and osteopontin in a manner involving activation of Piezo channels and the RhoA signaling pathway. Based on the methods used to stimulate MSCs in these studies, we believe it would be beneficial to translate these research findings to clinical applications if the bioreactors that generate these mechanical stimuli could be miniaturized and low-cost. Beyond a threshold amplitude or frequency, however, mechanical stimuli can harm cells, as they struggle to balance increasing levels of reactive oxygen species and inflammation [35]. Therefore, the practical application of these devices needs to be studied in more detail.

Kong et al. [90] showed that the nanotopography of TiO2 nanotubes promoted osteogenesis in BMSCs by increasing the nuclear localization of Yap and activating the expression of downstream Piezo1 and demonstrated that Piezo1 was a downstream effector of Yap-stimulated osteogenesis of BMSCs. Thus, the nanotopography of TiO2 nanotubes may promote the osteogenesis of BMSCs through the Yap/Piezo1 pathway. Interestingly, the results of this study complement the shortcomings of previous studies. Previous studies have generally shown that mechanical stimulation can induce the expression of Piezo channels, which then reactivate their signaling pathways to produce biological effects. Instead, these findings suggest that specific signaling pathways, such as the Yap/Taz signaling pathway, may regulate Piezo channel expression in MSCs after mechanical stimulation.

Furthermore, a study by Zhou et al. [91] showed that Piezo1 or more severe Piezo1/2 deficiency in mouse BMSCs resulted in multiple spontaneous fractures in neonatal mice due to the inhibition of osteogenic differentiation and increased bone resorption in BMSCs. These results suggest that although Piezo1 plays a major role in skeletal development, Piezo2 has a similar function to Piezo1 in BMSCs. In addition, the loss of Piezo1/2 in BMSCs in bone development and homeostasis can resist further bone loss caused by unloading [91]. Mechanistically, Piezo1/2 in BMSCs activates Ca^2+^ influx via fluid shear and ECM stiffness signaling, which stimulates calcium-regulated neurophosphatase (Calcineurin), thereby inducing the dephosphorylation of the transcription factors NFATc1, YAP1, and β-catenin and NFAT/YAP1/β-catenin complex formation to promote their synergistic activation [91]. These data suggest that the Ca^2+^/Ppp3ca signaling pathway is activated by Piezo1 and leads to the synergistic activation of Yap1, Ctnnb1, and Nfat, all of which act together to regulate the transcriptional changes that promote osteoblast differentiation and bone formation in BMSCs [91]. Thus, the synergistic activation of NFATc1, Yap1, and Ctnnb1 constitutes an integral mechanotransduction pathway that promotes bone formation.

### Piezo channels in dental-derived MSCs

Dental-derived MSCs (DMSCs) include dental pulp stem cells (DPSCs), periodontal ligament stem cells (PDLSCs), stem cells from exfoliated deciduous teeth (SCEDs), dental follicle stem cells (DFSCs), and stem cells from apical papillae (SCAPs) [26].

DPSCs were the first MSCs isolated from adult dental pulp and have potent self-renewal, proliferation, and differentiation capacity [92]. Gao et al. [59] showed that Piezo1 and Piezo2 were present in DPSCs and PDLSCs. Mousawi et al. [31] showed that in human dental pulp-derived MSCs (hDP-MSCs), the Piezo1 channel was activated to induce ATP release and subsequent P2 receptor purinergic signaling and downstream MEK/ERK and PYK2 signaling pathway activation to stimulate the migration of MSCs. Thus, the molecular and signaling mechanisms regulating MSC migration were revealed [31], which improves the comprehension of MSC migration and homing.

Low-intensity pulsed ultrasound (LIPUS) is an effective noninvasive treatment modality for accelerating fracture healing and hard tissue repair [93]. Ruthenium red (RR) is a Piezo ion channel blocker. RR significantly inhibited the proliferation of DPSCs induced by LIPUS stimulation but had no significant effect on the proliferation of PDLSCs [59]. RR may affect the MAPK signaling pathway in DPSCs and PDLSCs, and it has the most notable influence on ERK1/2/MAPK phosphorylation. Shen et al. [94] suggested that in PDLSCs, Piezo1 may deliver mechanistic signals through the ERK signaling pathway. RR significantly inhibited ERK1/2 activation by LIPUS in DPSCs [59], suggesting that the stimulation of DPSC proliferation by LIPUS involved Piezo-mediated regulation of the ERK1/2/MAPK signaling pathway. However, Hu et al. [32] showed that under inflammatory or noninflammatory conditions, LIPUS promoted endothelial differentiation and microangiogenesis in PDLSCs, whereas the Piezo1 inhibitor GsMTx4 inhibited the promoting effect of LIPUS. These experimental results suggest that Piezo1 may be involved in the effect of LIPUS on endothelial differentiation and angiogenesis in PDLSCs. Although MSCs are stimulated by LIPUS and activate the MAPK signaling pathway, MSCs derived from different tissues have different responses to LIPUS. For example, in these studies, LIPUS promoted the proliferation of DPSCs while promoting endothelial differentiation and angiogenesis in PDLSCs. These results suggest that the choice of tissue-derived MSCs is an important consideration when using MSCs for research or clinical applications. In addition, these findings illustrate the intricate and interacting signaling pathways downstream of Piezo channels, which requires further study to elucidate. LIPUS is an effective noninvasive therapeutic tool, and its clinical application to promote hard tissue repair and fracture healing may be promising.

Regarding PDLSCs, Jin et al. [33] showed a significant increase in the expression of Piezo1 and osteoclastogenesis-related markers in PDLSCs under compressive stress. When GsMTx4 was used to inhibit activation of the Piezo1 channel, the activity of the nuclear factor κB (NF-κB) signaling pathway was inhibited, which weakened the capacity of PDLSCs to induce osteoclast generation [33]. These results suggest that Piezo1 transduces and NF-κB signaling mediates mechanical stress-induced bone resorption [93]. Furthermore, a study by Wang et al. [95] showed that mechanical draft stress could promote the protein expression of Piezo1, which activated the Notch1 signaling pathway via Ca^2+^ as a second messenger, activating the expression of Runx2, ALP, BSP, and OCN and thereby promoting osteogenic differentiation in hPDLSCs. Plasmid-mediated overexpression of Piezo1 promoted hPDLSC osteogenic differentiation, which was blocked by the siRNA-Piezo1 interference plasmid [95]. Interestingly, although Piezo1 is a mechanosensitive receptor that can sense various types of mechanical stimulation, these results indicated that different types of mechanical stimulation could induce different and even opposite biological effects on MSCs. For example, compressive stress promotes the osteoclastogenic capacity of PDLSCs, while draft stress promotes the osteogenic capacity of PDLSCs. The intracellular signaling pathways involved in these processes are also different. This may be one of the mechanisms by which teeth can move within the alveolar bone during orthodontic procedures.

Miyazaki et al. [96] showed that in stem cells from human exfoliated deciduous teeth (SHED), HP noticeably promoted calcium deposition, the dentin-derived marker genes PANX3 and DSPP, and the WNT-related genes WNT5b and WNT16, as well as the nuclear translocation of RUNX2, while inhibiting SHED proliferation and enhancing primary cilia expression. Because PANX3, DSPP, WNT signaling, and the nuclear translocation of RUNX2 are essential markers of SHED differentiation into odontoblasts and play crucial roles in tooth development and dentin repair [97–101], these findings suggest that Piezo1 may act as a mechanosensor linking HP signals to intracellular signals during the differentiation of SHEDs into odontoblasts [96]. However, studies on bone development have shown that Wnt/β-catenin signaling positively regulates RUNX2 [96]. Therefore, further studies are needed to determine whether the nuclear translocation of RUNX2 is directly regulated by PIEZO1 signaling or indirectly induced by WNT expression.

DFSCs, which are derived from the follicles of unerupted teeth, are pluripotent and optimal stem cells for bone tissue engineering [102, 103]. A recent study showed that Piezo1 was activated by Yoda1 and significantly upregulated the mRNA and protein expression of ALP, RUNX2, OCN, and BMP2, as well as the expression of Wnt3a and β-catenin associated with the osteogenic pathway in DFCs. This finding suggests that the activation of Piezo1 can promote the proliferation and osteogenic differentiation of DFCs, which may be related to the Wnt/β-catenin pathway [104].

### Piezo channels in adipose-derived MSCs

ADSCs are critical MSCs. Huang et al. [105] showed that ADSCs adhering to UCST microgels could be stretched allosterically with microgel expansion, which upregulated TRPV4, Piezo1 channels, and phosphorylated ERK1/2 protein expression and increased intracellular calcium levels, ultimately promoting the differentiation of ADSCs toward nucleus pulposus-like cells. These results revealed that Piezo1 may affect the differentiation of ADSCs through the Piezo1-Ca^2+^-MAPK pathway [105]. These results suggest that microgel swelling-induced mechanical stimulation has great potential to regulate the differentiation of MSCs. Most current studies on mechanical stimulation are in vitro experiments performed in planar mode due to the need for external forces. However, the UCST microgels used in this study could swell due to temperature changes after entering the body to generate in situ mechanical stimulation to activate Piezo1 channels. The results of this study may be used to repair degenerated intervertebral disks in vivo. In another study on ADSCs, LIPUS could enhance the proliferation, cell cycle progression, and angiogenesis of ADSCs by activating the Piezo-ERK-VEGF pathway [34]. This study showed that ADSC transplantation paired with LIPUS could be used to treat diabetic erectile dysfunction synergistically [34].

### Piezo channels in human umbilical cord MSCs

A study by Sun et al. [106] showed that Piezo1 expression increased in human umbilical cord MSCs (hUC-MSCs) with increase in culture matrix stiffness, but Piezo2 expression was irregular. At 13~116 kPa matrix hardness but not 62~68 kPa, hUC-MSCs tended to differentiate more toward cardiomyocytes, which may be associated with the relatively low expression of Piezo1 and integrin β1 and Ca^2+^ concentrations in hUC-MSCs on a softer matrix [106]. Myocardial infarction is a cardiovascular disease with high mortality. Cardiomyocyte differentiation of hUC-MSCs may provide a replacement for cardiomyocytes damaged by MI.

### Piezo channels in human endometrial MSCs

In endometrial MSCs (EMSCs), long-term Piezo1 or store-operated Ca^2+^ entry (SOCE) activation has no cytotoxic effect, but it does slow their ability to migrate and proliferate. In addition, SOCE contributes to Piezo1-induced Ca^2+^ influx [107]. These data suggest that Piezo1 and SOCE are significant regulators of intracellular Ca^2+^, which may severely affect the migratory activity of EMSCs and may consequently alter their capacity for regeneration.

**Fig. 2:**
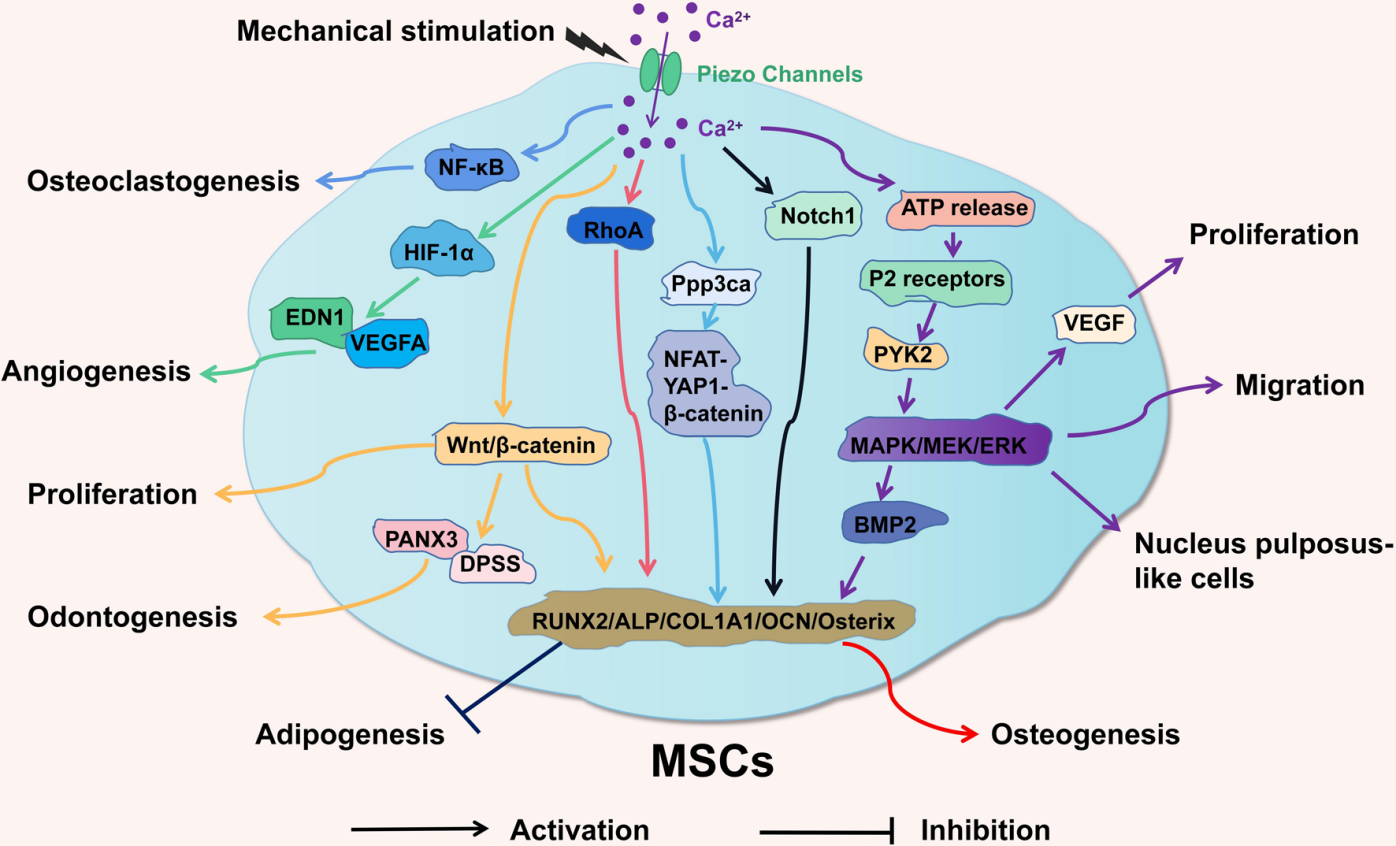
Schematic diagram of the effects of intracellular signaling pathways associated with Piezo channels on MSCs. Piezo channels are activated by mechanical stimulation, which causes the influx of Ca^2+^ into the cells and leads to the activation of multiple intracellular signaling pathways, producing a cascade of signals and ultimately regulating the functions of MSCs. This figure was created for this article, and the image depicted in this figure is ours; it is not based on any previously published image

To facilitate understanding, these key elements have been organized into two tables and a diagram (Tables 2, 3, and Fig. 2). Table 2 summarizes the effects of Piezo channels on the functions of MSCs based on the classification of MSCs from different tissues. Table 3 summarizes the corresponding mechanisms and signaling pathways based on the classification of different functional effects of Piezo channels on MSCs.

**Table 2:** The functional roles of Piezo ion channels in MSCs

Ion channel	Cell/tissue	External stimulation	Signaling pathway	Functional effect	References
Piezo1	BMSCs	HP	Piezo1- ERK1/2, p38 MAPK- BMP2	Promote osteogenesis and inhibit adipogenesis in BMSCs	[28]
Piezo1	BMSCs	Viscoelasticity of hydrogels	-	Regulate the differentiation of BMSCs from osteogenesis to adipogenesis	[29]
Piezo1	BMSCs	Triboelectric stimulation	Piezo1-Ca^2+^-HIF-1α-EDN1, VEGFA	Restore the vitality of aging BMSCs and enhance osteogenesis and proangiogenic functions	[30]
Piezo1	MSCs	Antibody of Piezo1	-	Enhance osteogenesis in MSCs	[36]
Piezo1/2	BMSCs	Nanovibrational stimulation	-	Enhance three-dimensional osteogenesis in BMSCs	[37]
Piezo1/2	BMSCs	Nanovibrational stimulation	Piezo1/2-ERK1	Enhance the osteogenic differentiation of BMSCs	[35]
Piezo1/2	BMSCs, ADSCs, UCSCs	Megahertz-order nanomechanostimulation	Piezo1/2-RhoA	Enhance the osteogenic differentiation of MSCs	[89]
Piezo1	BMSCs	Nanotopography	Yap-Piezo1	Enhance osteogenesis in BMSCs	[90]
Piezo1/2	BMSCs	Fluid shear stress and ECM stiffness	Piezo1/2-Ca^2 +^ -Pppp3ca-NFATc1, YAP1, Ctnnb1	Promote osteogenic differentiation and bone formation in BMSCs	[91]
Piezo1/2	DPSCs	LIPUS	Piezo1/2-ERK1/2, MAPK	Enhance the proliferation of DPSCs	[59]
Piezo1	DPMSCs	Yoda1	Piezo1-PYK2, MEK/ERK, P2 receptor purinergic signaling	Regulate the migration of DPMSCs	[31]
Piezo1	PDLSCs	GsMTx4	Piezo1-ERK	Suppress key PDLSCs biomarkers	[94]
Piezo1	PDLSCs	LIPUS	–	Promote endothelial differentiation and angiogenesis in PDLSCs	[32]
Piezo1	PDLSCs	Compressive stress	Piezo1-NF-kB	Enhance the ability of PDLSCs to induce osteoclastogenesis	[33]
Piezo1	PDLSCs	Stretch stress	Piezo1-Notch1	Enhance osteogenic differentiation in PDLSCs	[95]
Piezo1	SHEDs	HP	Piezo1-Wnt/β-catenin	Enhance osteogenesis and odontogenesis in SHEDs	[96]
Piezo1	DFSCs	Yoda1	Piezo1-Wnt/β-catenin	Promote the proliferation and osteogenic differentiation of DFCs	[104]
Piezo1	ADSCs	Swelling of microgels	Piezo1-Ca^2+^-MAPK	Promote the differentiation of ADSCs into nucleus pulposus-like cells	[105]
Piezo1/2	ADSCs	LIPUS	Piezo1/2-ERK- VEGF	Promote the proliferation and cell cycle progression of ADSCs	[34]
Piezo1	UCMSCs	Stiffness	Piezo1-Ca^2+^-	Promote the differentiation of UCMSCs into cardiomyocytes in a low stiffness matrix	[106]
Piezo1	EMSCs	Yoda1	Piezo1-Ca^2+^-	Inhibit the migration and proliferation of EMSCs	[107]

**Table 3:** The functional effects and signaling pathways of Piezo channels in MSCs

Functional effect	Signaling pathway	Ion channel	References
**+ **Proliferation	Piezo1/2-ERK1/2, MAPK;	Piezo1/2	[34, 59, 104]
	Piezo1-Wnt/β-catenin;		
	Piezo1/2-ERK- VEGF		
− Proliferation	Piezo1-Ca^2+^	Piezo1	[107]
**+ **Osteogenesis	Piezo1- ERK1/2, p38 MAPK- BMP2;	Piezo1/2	[28, 30]
	Piezo1-Ca^2+^-HIF-1α-EDN1, VEGFA;		[36, 37]
	Piezo1/2-ERK1;		[35, 89]
	Piezo1/2-RhoA;		[90, 91]
	Yap-Piezo1;		[95, 96]
	Piezo1/2-Ca^2+^-Pppp3ca-NFATc1, YAP1,		[104]
	Ctnnb1;		
	Piezo1-Notch1;		
	Piezo1-Wnt/β-catenin;		
**+ **Adipogenesis	–	Piezo1	[29]
− Adipogenesis	Piezo1- ERK1/2, p38 MAPK- BMP2;	Piezo1	[28]
**+ **Migration	Piezo1-PYK2, MEK/ERK, P2 receptor purinergic signaling	Piezo1	[31]
− Migration	Piezo1-Ca^2+^-	Piezo1	[107]
**+ **Angiogenesis	Piezo1-Ca^2+^-HIF-1α-EDN1, VEGFA	Piezo1	[30, 32]
**+ **Osteoclastogenesis	Piezo1-NF-kB	Piezo1	[33]
**+ **Odontogenesis	Piezo1-Wnt/β-catenin	Piezo1	[96]
**+ **NP-like cells	Piezo1-Ca^2+^-MAPK	Piezo1	[105]
**+ **Restore vitality	Piezo1-Ca^2+^-HIF-1α-EDN1, VEGFA	Piezo1	[30]

“+” indicates promotion, and “−” indicates inhibition

## Conclusion and future directions

Studies have shown that Piezo channels play crucial roles as mechanosensors in the proliferation, differentiation, and migration of MSCs. In MSCs, various mechanical stimuli and Piezo channel activators can activate Piezo channels, which in turn activate a variety of intracellular signaling pathways, thereby regulating the behavior of MSCs. These behaviors include proliferation, migration, osteogenic differentiation, lipogenic differentiation, endothelial differentiation, osteoblast differentiation, odontogenic differentiation, nucleus pulposus-like cell differentiation, and cardiomyocyte differentiation.

However, as summarized in Table 3, it is clear that although these factors all activate Piezo1 channels, different research teams have come up with opposite results, such as opposing effects on proliferation, adipogenesis, and migration. The reasons for these differences may be that Piezo signaling interacts with other signaling pathways in the cell, MSCs derived from different tissues have their own differentiation preferences, or experimental errors of the different research teams. If we want to determine the causes of these problems, more detailed, deeper, and standardized research is essential. However, the results showing that activation of Piezo channels can promote osteogenesis and angiogenesis of MSCs are consistent in different studies. Moreover, current studies on the effects of Piezo channels on MSCs mainly focus on osteogenesis. In fact, the effects of Piezo channels on angiogenesis, odontoblasts, migration, and other biological functions of MSCs are promising and worthy of further exploration.

The latest studies on the function of Piezo channels in MSCs are fascinating and promising, and for a deeper comprehension of the role and purpose of Piezo channels in MSCs, numerous issues need to be resolved. The role of Piezo1 in MSCs has been extensively studied, but the role of Piezo2 in MSCs remains unclear. Do Piezo1 and Piezo2 play a dominant role in MSCs? Do Piezo1 and Piezo2 have different roles in MSCs of different tissue origins? Given the stable expression and biological role of Piezo channels in MSCs of different tissue origins, how should Piezo channels in MSCs be specifically targeted to provide a new way to treat the corresponding diseases? How can the in vivo retention of transplanted MSCs be enhanced? Piezo1, Piezo2, and Piezo1/Piezo2 gene mutations (knockout or knock-in) in specific MSCs as well as experimental mouse models or cellular models may need to be established to provide answers to these questions [8]. The answers to these questions will contribute to a deeper comprehension of the role of Piezo channels in MSCs and facilitate the clinical application of MSCs.

## Data Availability

Not applicable.

## Abbreviations

MSCs: Mesenchymal stem cells
CTD: Carboxy-terminal structural domain
CED: Carboxy-terminal extracellular domain
IH: Inner helix
OH: Outer helix
PHs: Peripheral helices
GsMTx4: Grammostola spatulata mechanotoxin 4
ECM: Extracellular matrix
BMSCs: Bone marrow-derived mesenchymal stem cells
HP: Hydrostatic pressure
ERK: Extracellular signal-regulated kinase
MAPK: Mitogen-activated protein kinase
MEK: Mitogen-activated protein kinase
BMP2: Bone morphogenetic protein 2
ALP: Alkaline phosphatase
COL1A1: Collagen, type I, alpha 1
WP-TENG: Wearable pulsed triboelectric nanogenerator
Runx-2: Runt-related transcription factor 2
OCN: Osteocalcin
HIF: Hypoxia-inducible factor
EDN: Endothelin
VEGF: Vascular endothelial growth factor
GO-MNPs: Graphene oxide-based nanocomposites
TRP: Transient receptor potential
KCNK: Potassium channel, subfamily K
ADSCs: Adipose derived stem cells
UCSCs: Umbilical cord blood-derived stem cells
RhoA: Ras homolog gene family, member A
YAP: Yes-associated protein
Pppp3ca: Serine/threonine phosphatase calcineurin
NFAT: Nuclear factor of activated T cells
Ctnnb1: Catenin beta 1
DMSCs: Dental-derived mesenchymal stem cells
DPMSCs: Dental pulp-derived mesenchymal stem cells
PDLSCs: Periodontal ligament stem cells
SCEDs: Stem cells from exfoliated deciduous teeth
SCAPs: Stem cells from apical papillae
LIPUS: Low-intensity pulsed ultrasound
PYK2: Proline-rich tyrosine kinase 2
RR: Ruthenium red
NF: Nuclear factor
BSP: Bone sialoprotein
PANX3: Pannexin 3
DSPP: Dentin sialophosphoprotein
SHED: Stem cells from human exfoliated deciduous teeth
DFSCs: Dental follicle stem cells
UCST: Upper critical temperature
UCMSCs: Umbilical cord mesenchymal stem cells
EMSCs: Endometrial mesenchymal stem cells
SOCE: Store-operated Ca^2+^ entry
